# Multiplication rate variation in the human malaria parasite *Plasmodium falciparum*

**DOI:** 10.1038/s41598-017-06295-9

**Published:** 2017-07-25

**Authors:** Lee Murray, Lindsay B. Stewart, Sarah J. Tarr, Ambroise D. Ahouidi, Mahamadou Diakite, Alfred Amambua-Ngwa, David J. Conway

**Affiliations:** 1 Pathogen Molecular Biology Department, London School of Hygiene and Tropical Medicine, Keppel Street, London, WC1E 7HT United Kingdom; 2 Le Dantec Hospital, Universite Cheikh Anta Diop, Dakar, Senegal; 3 Malaria Research and Training Center, University of Bamako, Bamako, Mali; 4 Medical Research Council Unit, Fajara, The Gambia

## Abstract

It is important to understand intrinsic variation in asexual blood stage multiplication rates of the most virulent human malaria parasite, *Plasmodium falciparum*. Here, multiplication rates of long-term laboratory adapted parasite clones and new clinical isolates were measured, using a newly standardised assay of growth from low starting density in replicate parallel cultures with erythrocytes from multiple different donors, across multiple cycles. Multiplication rates of long-term established clones were between 7.6 and 10.5 fold per 48 hours, with clone Dd2 having a higher rate than others (clones 3D7, HB3 and D10). Parasite clone-specific growth was then analysed in co-culture assays with all possible heterologous pairwise combinations. This showed that co-culture of different parasites did not affect their replication rates, indicating that there were no suppressive interactions operating between parasites. Multiplication rates of eleven new clinical isolates were measured after a few weeks of culture, and showed a spectrum of replication rates between 2.3 and 6.0 fold per 48 hours, the entire range being lower than for the long-term laboratory adapted clones. Multiplication rate estimates remained stable over time for several isolates tested repeatedly up to three months after culture initiation, indicating considerable persistence of this important trait variation.

## Introduction

The malaria parasite *Plasmodium falciparum* is responsible for up to half a million human deaths each year^1^, but there are basic features of its biology that remain poorly understood. As the density of parasites is a primary determinant of disease, identifying if there is intrinsic variation in parasite multiplication rates in the blood is important from a clinical as well as evolutionary perspective^2^. Although very high parasite densities are associated with severe disease^3^, a larger number of infections are clinically mild, and sensitive detection methods reveal most infections to be asymptomatic with very low parasite densities^4^. Analyses of *in vivo* growth rate of *P. falciparum*, using historical data from induced-malaria treatments of neurosyphillis cases, indicated some variation in multiplication rates among three parasite strains, with an overall average estimate of approximately 8 fold per 48 hour period^5^, although under alternative modelling parameters this could be slightly higher^6^. Recent studies of controlled human infections with *P. falciparum* clone 3D7 used in vaccine challenge studies also indicate a similar multiplication rate^7^. Although virulence may not be experimentally analysed in human infections, studies using rodent malaria parasites indicate that strain-dependent variation in parasite multiplication rate is associated with disease in mice^2, 8^.

Separate studies of *P. falciparum* clinical samples cultured through the first cycle *ex vivo* have indicated variation in multiplication rates, although these are generally lower than expected on the basis of the *in vivo* studies. Median multiplication rates of parasites from uncomplicated clinical malaria cases are reported as being 2.9-fold in a study in Thailand, 3.1-fold in Mali, 2.3-fold in Kenya, and approximately 3.3-fold in Uganda^9–11^. Parasites from severe malaria cases in Mali and Kenya had similar multiplication rates as those from uncomplicated cases^10^, whereas parasites from severe malaria cases in Thailand and Uganda were reported to have higher multiplication rates on average^9, 11^. A limitation of these studies is that they were based on only a single measurement per isolate, so they lack technical or biological replication. Additionally, assessment of parasite multiplication phenotypes in the first cycle *ex vivo* may be confounded by heterogeneous parasite viability or erythrocyte condition among individual patients at the time of sampling, due to variable processing and storage of samples, or to physiological states including anaemia^12^.

Parasite multiplication rates have been assessed for only a few laboratory adapted clones of *P. falciparum* ^13^, and from genetic cross progeny of two clones^14^, but these may not be representative of natural parasite populations. Many parasites that have been in culture for a long time are loss-of-function mutants, with particular genes having acquired premature stop codons^15^, or having been lost in subtelomeric deletions^16^. Determining the true range of variation in asexual multiplication rate of *P. falciparum* would be an important step in being able to identify determinants of growth and virulence factors. A feature of malaria infections in endemic regions is the presence of multiple distinct parasite clones within a proportion of individual hosts^17, 18^. Studies on rodent malaria models have given contrasting conclusions as to whether competitive suppression occurs between different co-infecting genotypes^19, 20^, and co-culture studies on *P. falciparum* have not yielded consistent findings on interactions between different clones^21–23^. It remains unknown how malaria parasites would be able to detect the presence of other clones within a multi-clone infection, or within heterologous co-cultures, although potential intercellular communication mechanisms are suggested^24, 25^.

A reliable assay is here described for quantifying *P. falciparum* asexual blood stage multiplication rates in a standardised manner over multiple cycles, using biological triplicate measurements in different erythrocytes. This is applied to measure the multiplication rate variation among unrelated laboratory clones cultured separately, and in mixed co-cultured combinations, as well as the multiplication rates of a panel of clinical isolates from three endemic countries.

## Results

### Quantifying multiplication rates of *P. falciparum* laboratory clones

**Figure 1:**
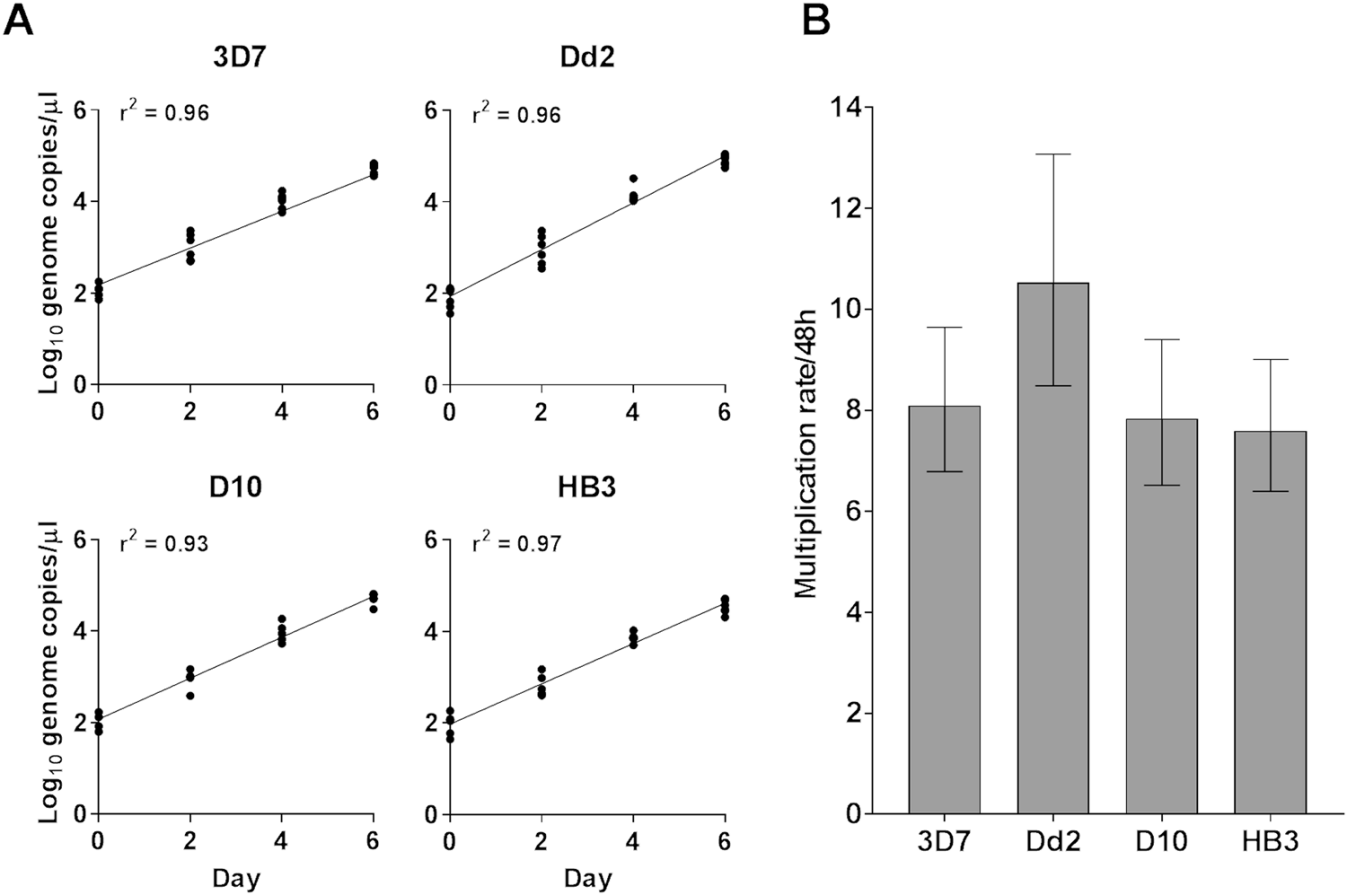
Measurement of exponential multiplication rates of laboratory adapted clones of *P. falciparum*. (**A**) 6-day growth assays for four laboratory clones, showing parasite density as numbers of genome copies per microlitre (on a log_10_ scale). Six biological replicates were performed using different donor erythrocytes. Estimation of parasite copy numbers by quantitative PCR was confirmed to be highly correlated with counts of parasites by microscopy of Giemsa-stained slides (Supplementary Figure S1). ‘3D7’ here refers to the genetically modified clone 3D7-HT-GFP^35^ whereas the other clones were not genetically modified. (**B**) General linear model estimates of the replication rates per 48 hours over the 6-day assays for each of the four laboratory clones (point estimates with 95% confidence intervals).

Multiplication rates of four unrelated laboratory clones (Dd2, D10, HB3 and 3D7-HT-GFP) were measured, by analysing a six-day period of exponential growth from a starting inoculum density of 0.02% infected erythrocytes. Assays were conducted on each clone in parallel using six biological replications, with different donor erythrocytes in separate flasks. Parasite density in each flask on days 0, 2, 4 and 6 was quantified by qPCR measurement of parasite genome numbers per microlitre in sampled aliquots, and plotted on a log_10_ scale (Fig. 1A). This showed strong correlation with parasite numbers estimated by microscopy count data (Pearson’s r = 0.93, p-value < 1 × 10^−10^; Supplementary Fig. S1A), with marginally higher sensitivity achieved by qPCR (Supplementary Fig. S1B) as seen in a previous comparison^26^. Generalized linear modelling, with high coefficients of determination across all the biological replicates for each clone (*r* ^2^ > 0.95), yielded estimated multiplication rates per 48 hour period over the 6 day assay (Fig. 1B). Clone Dd2 had a higher multiplication rate (10.53 fold, 95% CI 6.79–13.07) than HB3 (7.59 fold, 95% CI 6.40–9.00), D10 (7.83 fold, 95% CI 6.52–9.41) and 3D7-HT-GFP (8.09 fold, 95% CI 6.79–9.64).

### Clone-specific multiplication rates in heterologous *P. falciparum* co-cultures

**Figure 2:**
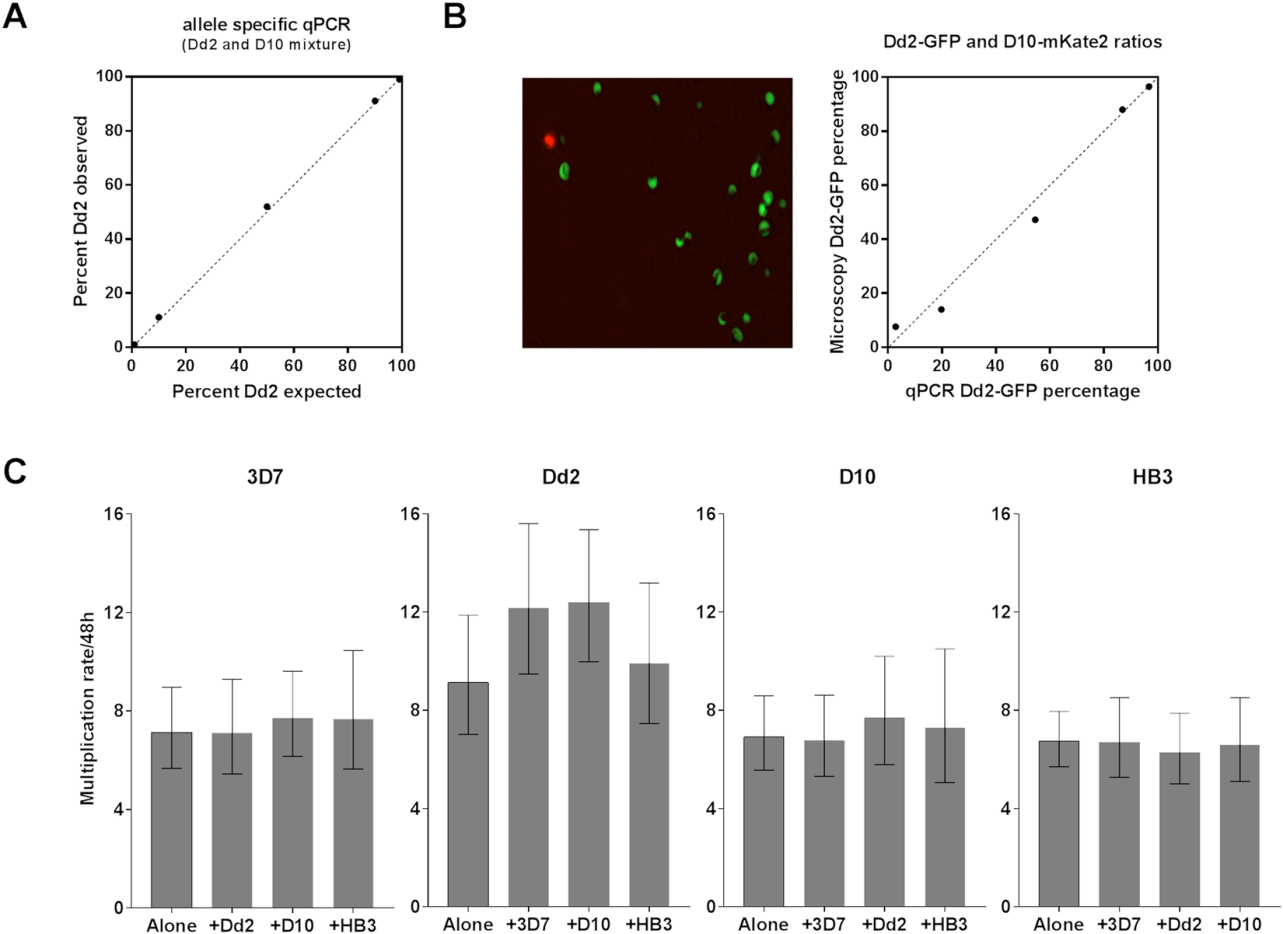
Quantitation of parasite multiplication rates for four laboratory clones across three intraerythrocytic cycles for all pairwise co-cultured combinations. (**A**) Allele-specificity of qPCR assays for clone-specific parasite density measurement. Three different allele-specific sets of assays were used to analyse all pairwise combinations of four *P. falciparum* laboratory clones. Each assay was first evaluated in a comparison of observed versus expected ratios for a pair of heterologous clones. The assay shown here is described in this paper, and discriminates *msp6* alleles to accurately quantify Dd2 and D10 DNA mixed at different ratios from 1:99 to 99:1. Two different assays to discriminate *msp1* alleles, used to quantify parasite DNA in other heterologous mixtures (Supplementary Figure S2), have been previously described^21, 22^. (**B**) Comparison of ratios of different parasite clones in mixtures assayed by fluorescence microscopy and allele-specific qPCR. There is a high correlation between the independent measurements (*r* ^2^ = 0.984) across a range of ratios from 1:20 to 20:1 for the Dd2-GFP and D10-mKate2 clonal mixtures. (**C**) *P. falciparum* clone-specific parasite multiplication rates (with 95% confidence intervals) are similar in monoculture and pairwise co-cultured combinations. ‘3D7′ here refers to the genetically modified clone 3D7-HT-GFP^35^. The allele-specific qPCR assays of parasite densities (at days 0, 2, 4 and 6) yield estimates of clone-specific growth per 48 hour period by general linear modelling of exponential growth over six days (values are listed in Supplementary Table S1).

To test for potential interactions between different *P. falciparum* clones during co-culture, assays were performed for all six pairwise combinations of the four laboratory clones. Each of three allele-specific PCR assays required for measuring clone-specific growth in the co-cultures was first carefully validated. The proportions of genome copies measured for each parasite clone was highly concordant with the known proportions throughout the range of heterologous DNA mixed ratios from 1:99 o 99:1 (*r* ^2^ values > 0.99 for each pairwise combination, Fig. 2A and Supplementary Fig. S2). To confirm that clone-specific proportions were faithfully measured from DNA extracted, a spectrum of ratios from 1:20 to 20:1 of two fluorescent parasite clones (Dd2-GFP and D10-mKate2) were measured using the quantitative allele-specific PCR assays and compared to direct live imaging fluorescent microscopy counting, confirming a high level of concordance between the methods (*r* ^2^ values > 0.98, Fig. 2B).

All possible heterologous co-culture pairwise combinations at 1:1 ratios (each clone at a starting density of 0.01% infected erythrocytes) were tested for multiplication rates in triplicate flasks with different donor erythrocytes. Allele-specific measurement of parasite genome copy numbers was performed on samples from the cultures at days 0, 2, 4 and 6, and multiplication rates per 48 hours derived by generalized linear modelling (Fig. 2C). For each of the clones, this allele-specific analysis of *P. falciparum* multiplication rates for the monoculture controls yielded similar estimates to those obtained in the independent experiments reported above using qPCR assays based on conserved sequences. Under all conditions, clone Dd2 had a higher multiplication rate than any of the other three clones. In comparison with monoculture controls, there was no reduction in multiplication rates seen in any parasite clone in any of the co-culture mixtures (Fig. 2C and Supplementary Table S1), indicating that there was no suppression due to heterologous interactions.

### Multiplication rates of *P. falciparum* clinical isolates

Multiplication rate assays were performed on eleven new *P. falciparum* clinical isolates, following at least two weeks of culture after isolation so that parasites were growing in erythrocytes from anonymous heterologous donors. Microsatellite genotype profiling of four loci confirmed that all isolates were genotypically distinct except for two of the Senegalese isolates (SEN215 and SEN232, cultured at separate times, Supplementary Table S2). Three isolates (GUI246, MAL250, and SEN251) clearly contained multiple genotypes while each of the others contained a single predominant genotype (Supplementary Table S2). Parasite multiplication was determined from a starting density of 0.02% infected erythrocytes, after diluting into triplicate flasks with different donor erythrocytes under the same protocol as used for laboratory adapted clones. Analysis of parasite densities over a six-day period yielded lower coefficients of determination for multiplication rates of these clinical isolates (mean *r* ^2^ = 0.89, range from 0.76 to 0.99) than had been seen with assays of the laboratory adapted clones reported above, and inspection of the data showed this was due to a lower multiplication rate after day 4 of the assay in several of the isolates (Supplementary Fig. S3). As the coefficient of determination values for the first 4 days of the assay were significantly higher (mean *r* ^2^ = 0.94, range from 0.88 to 0.99), these data were used to estimate the multiplication rates per 48 hours.

**Figure 3:**
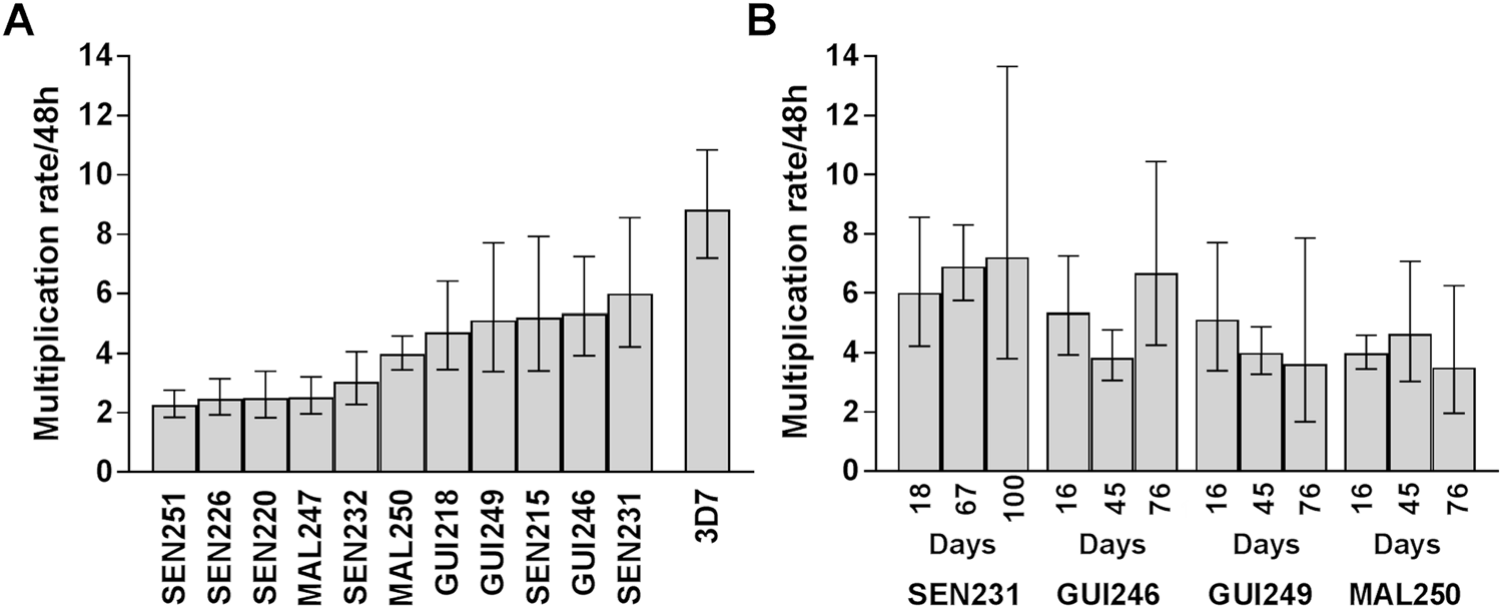
Parasite multiplication rates (with 95% confidence intervals) of new *P. falciparum* clinical isolates, sampled from patients in three West African countries (GUI, Guinea; SEN, Senegal, MAL, Mali). (**A**) Eleven isolates were analysed after growth in culture for at least two weeks (between 16 and 32 days) prior to assay. Exponential growth was measured over 4 days using a general linear model approach. All assays were conducted with biological triplicates in separate flasks with different donor erythrocytes, except for SEN232 which was assayed in duplicate. Six of the isolates (SEN232, GUI246, MAL247, GUI249, MAL250, SEN251) were assayed in medium supplemented by Albumax and serum, whereas the other five were assayed by medium with Albumax alone as noted in the Materials and Methods (results were similar for the two groups, Mann-Whitney test P = 0.90). As a control, the laboratory adapted clone 3D7-HT-GFP (here labelled ‘3D7′) was tested in parallel in each assay, yielding a consistent multiplication rate of approximately 8 fold per 48 hours. (**B**) Four of the clinical isolates cultured for longer periods were assayed at three different time points after culture initiation (up to 76 or 100 days for each isolate). These showed no significant changes in multiplication rate over time in culture. Assays were conducted with biological triplicates, except for the last cultured time point for which the assay was conducted in duplicate.

The multiplication rates of the clinical isolates ranged from 2.26 to 6.00-fold, with a mean of 3.92-fold (Fig. 3A). In contrast, parasite clone 3D7-HT-GFP which was tested in parallel on all occasions had a consistently higher multiplication rate close to 8.0-fold, similar to that seen in all previous assays. Variation in multiplication rates of clinical isolates did not depend on whether they had single or multiple detected genotypes (means of 3.94 and 3.86 fold respectively; Mann-Whitney test, P = 0.92). The multiplication rate variation also did not associate with use of alternative media supplements (mean of 3.71 fold for isolates grown with Albumax supplementation, and mean of 4.17 fold for isolates grown with Albumax and serum; Mann-Whitney test, P = 0.90).

As all eleven clinical isolates had lower multiplication rates than all four laboratory clones tested (Mann Whitney test, P < 0.001), it was considered that faster multiplication may be a trait selected during the process of culture adaptation. Although the length of time in culture of the eleven isolates prior to initial assay (between 16 and 37 days) was not correlated with multiplication rate (Spearman’s ρ = 0.03, P = 0.92), to test for changes over a longer period four of the isolates were cultured for up to a few months and assayed at two additional time points. No significant changes in multiplication rate were detected over the periods investigated (Fig. 3B). The four isolates had a mean 48 hour multiplication rate of 5.11-fold at the first time point (16 or 18 days after culture initiation) and 5.25-fold at the last time point (76 or 100 days after culture initiation).

## Discussion

Substantial intrinsic variation in *P. falciparum* multiplication rates is revealed, with long-term laboratory adapted lines multiplying between 7.6 and 10.5 fold every 48 hours and clinical isolates that have been cultured for only a few weeks multiplying between 2.3 and 6.0 fold every 48 hours. Previously reported multiplication rates for clinical malaria isolates involved single *ex vivo* assay measurements during the first cycle *ex vivo* ^9–11^, and yielded slightly lower average multiplication rate estimates compared with those measured for the clinical isolates here. Lack of biological replicates and variable viability of parasites in initial clinical samples are concerns for first cycle assays,, whereas the assays described here involved quantitation over multiple cycles and assay replicates using unrelated donor erythrocytes, to enable more robust comparisons of parasite multiplication rate phenotypes.

There were no significant short-term modifications in the multiplication rate of any parasite clone caused by co-culture with heterologous clones, under the assay conditions allowing exponential growth from a low starting density. Although inter-clonal suppressive effects might occur at higher densities in culture, previous attempts to investigate this have yielded variable results. One study suggested competitive suppression of clone HB3 by clone Dd2, based on endpoint density measurements after a short period of co-culture^21^, while another showed Dd2 to have a more consistent average growth rate only slightly higher than that of HB3 in longer-term co-culture^23^. Another study indicated the general difficulty in separating any genotype-specific effects from those determined by total parasite density^22^. A recent study comparing two different clones has indicated differences in their density dependence when cultured separately^27^, a heterogeneity which would complicate the interpretation of co-culture at high densities.

Several different components of the parasite developmental cycle may potentially influence multiplication rates, including intraerythrocytic cycle duration, numbers of merozoites per schizont, and erythrocyte invasion efficiency^9, 28^. A previous comparison of laboratory clones Dd2 and HB3 estimated that Dd2 had a shorter mean cycle time, a higher mean number of merozoites per schizont, and higher merozoite invasion efficiency^13^. Therefore, each of the measured parameters could contribute to the higher replication rate for Dd2 compared to HB3 that has been confirmed here. The potential contribution of these different parameters has yet to be determined for other laboratory clones or clinical isolates. The possible effects of host erythrocyte variation have been minimised in the current study by assaying each of the parasite lines in erythrocytes from multiple different anonymous donors as biological replicates. As erythrocyte variation can potentially affect parasite multiplication, it would be particularly interesting to test the relative multiplication rates of different parasite isolates in erythrocyte types associated with protection from severe malaria in African populations, including locally selected glycophorin and haemoglobin variants^29, 30^.

Although multiplication rates were measured at multiple time points for several of the clinical isolates that were continued in culture for a few months, no significant changes were seen. It is likely that culture adaptation occurs over longer time scales, requiring sufficient numbers of generations to allow emerging adaptive mutants to go to high frequencies^15^. It may be expected that epigenetic regulation of clonally variable transcriptional variation could allow phenotypic change to occur more rapidly^31^, but expression profiles have not been characterised in clinical isolates during the process of culture adaptation, aside from analyses of candidate genes such as the highly transcriptionally variable *var* genes^32^ and merozoite invasion ligand genes^33^ in a small number of isolates.

Multiplication rate measurements are more robust when conducted over more than a single cycle, thereby requiring a minimum of four days to cover at least two of the 48 hour periods corresponding to an average replication cycle of this parasite species. Although laboratory adapted parasite clones showed consistent multiplication rates throughout a six day growth assay, whether in monoculture or in mixed heterologous co-cultures, the clinical isolates showed more consistent growth during the first four days. As several of the clinical isolates showed slower growth later in the assay, which would have artificially lowered the multiplication rate estimates, analysis of multiplication over four days was more accurate, with higher coefficient of determination. Although data with lower measurement coefficients have previously been included for logistic multiplication rate estimation in induced infections^34^, and culture assays^23^, a high threshold for inclusion such as applied here enables focus of analysis on more precise data. Study of heterogeneous samples can thereby be prospectively conducted with similarly tight assay replication, which would enable future comparisons of parasites from very low parasitaemia asymptomatic infections, or from high parasitaemia severe infections.

## Materials and Methods

### *P. falciparum* laboratory adapted clones and new clinical isolates

For monoculture and competitive growth studies, four different *P. falciparum* laboratory adapted clones were studied that had been originally derived from clinical infections many years ago: Dd2 (from the area of Southeast Asia formerly known as ‘Indochina’), HB3 (from Honduras), D10 (from Papua New Guinea), and 3D7 (from a case of ‘airport malaria’ in the Netherlands with a parasite type having genetic similarities to African *P. falciparum* isolates). For 3D7, a genetically modified sub-clone was used that had been previously engineered to express the green fluorescent protein (3D7-HT-GFP kindly provided by Professor Bob Sinden)^35^. This clone was used throughout as a control in the multiplication rate assays. These four laboratory-adapted lines were each cultured from pre-existing stocks and were not re-cloned prior to this study.

*P. falciparum* clinical isolates were sampled from patients who tested positive for malaria by immunochromatic rapid diagnostic testing and who had reported not taking antimalarial drugs during the preceding 3 days. Patients presented at local health facilities at three different sites in West Africa (Faranah in the Republic of Guinea in 2012, the Pikine area of Dakar in Senegal in 2013, and Nioro du Sahel in Mali in 2014), and written informed consent for blood sampling for the study was obtained from patients or their parents or guardians. Prior to antimalarial treatment, venous blood samples of up to 5 ml in volume were collected into anticoagulant tubes and centrifuged, following which plasma and leukocyte buffy coats were removed, and erythrocytes were cryopreserved in glycerolyte at −80 °C or in liquid nitrogen until shipment on dry ice to the London School of Hygiene and Tropical Medicine where culture was performed. Eighteen isolates (five from Guinea, nine from Senegal, and four from Mali) were selected for testing in this study after they had been cultured for at least two weeks, by which time the majority of erythrocytes had been replaced by those from non-patient donors. Isolates were given laboratory codes within a single series, so that laboratory workers were blinded to the origin of each isolate and the assays were conducted on parasites from different populations over the same period, as generally recommended for assays of clinical isolate phenotypes^36^. High quality assay data with sufficient biological and technical replication were achieved for eleven of the isolates (three from Guinea, six from Senegal and two from Mali) following all the procedures described below. Approval of the study was granted by the Ethics Committee of the London School of Hygiene and Tropical Medicine, the National Ethics Committee for Health Research in the Republic of Guinea, the Ethics Committee of the Ministry of Health in Senegal, and the Ethics Committee of the Ministry of Health in Mali. Under approval of the Ethical Committees, all methods were performed in accordance with the relevant guidelines and regulations of the participating research institutes.

### Parasite culture and multiplication rate assays

Parasites were cultured at 37 °C in anonymous adult donor erythrocytes at 3% haematocrit in RPMI 1640 medium supplemented with 0.5% Albumax II (Life Technologies, Paisley, United Kingdom), or in particular assays with 0.3% Albumax II and 2% human AB serum (GE Healthcare, Amersham, United Kingdom), under an atmosphere of 5% O_2_, 5% CO_2_, and 90% N_2_. Multiplication rate assays were conducted with up to six biological replicates, using parallel culture flasks with each flask containing 10 mL culture using erythrocytes from a different anonymous adult donor of unknown blood group. Blood from multiple different donors, all adult volunteers living in the UK and working at LSHTM, was collected the day prior to the start of each multiplication rate assay, and erythrocytes were washed in RPMI 1640 incomplete medium and stored at 4 °C at 50% haematocrit. Parasite cultures were asynchronous prior to starting the assay, and each replicate was initiated at a parasitaemia of 0.02% by dilution of an initial culture of at least 0.2% parasitaemia into fresh donor erythrocytes (dilutions were a minimum of 10 fold so that at least 90% of erythrocytes within each replicate of the assay were from different anonymous donors). During the 6-day assay, every 48 hours (day 0, 2, 4, and 6) a 300 µL sample of resuspended culture medium was taken, from which 100 μL was pelleted and used to make a slide for giemsa staining and microscopy, with the remaining 200 μL frozen at −20 °C for DNA extraction and qPCR. Fresh complete RPMI medium change was performed on days 2, 4, and 5. Of the eleven clinical isolates for which high quality assay data were obtained for analysis, six isolates (SEN232, GUI246, MAL247, GUI249, MAL250, SEN251) were grown and assayed in RPMI medium with 0.5% Albumax II as for the laboratory adapted isolates, while the remaining five isolates were assayed in RPMI medium with 0.3% Albumax II and 2% human serum.

DNA from each culture was extracted using a DNA Blood Mini kit (Qiagen), eluted in 40 μL and stored at −20 °C. Genomic DNA standard controls were run in technical triplicates for each qPCR run, with a concentration range from 1 million down to 10 copies μL^−1^, and DNA extracted from each biological replicate at each timepoint was also assayed in technical triplicate and alongside negative template control samples. Primers targeting the highly conserved *Pfs25* locus^37^ were used at 300 nM final concentration with SYBR select master mix (Applied Biosystems) in a total reaction volume of 10 μL, for qPCR assays run on a Rotor-Gene 3000 machine (Corbett Life Sciences and Qiagen), with cycling conditions of 50 °C for 2 minutes, 95 °C for 2 minutes, 40 cycles of 95 °C for 15 seconds and 60 °C for 1 minute. Following each machine run, a melt curve was performed, to confirm specificity of the qPCR amplicons. Quality control filtering of the raw data involved removal of measurements that were ≥0.5 log genome copies different from other replicates for that individual clone. To ensure focus on high quality data, an isolate was dropped from analysis if there was more than one missing biological replicate data point at any time point, or if the final time point measurement did not have a mean of at least 500 genome copies measured in each replicate. This filtering enabled analysis of eleven (61%) of the eighteen clinical isolates for which the assay was initially attempted.

### Design and validation of allele-specific qPCR assays

For allele-specific primer design, DNA sequences for several polymorphic candidate genes of four laboratory clones (Dd2, HB3, D10 and 3D7) were visualised in Jalview 2.8.1^38^. Two previously described allele-specific quantitative PCR assays which targeted the *msp1* gene^21, 39^ were suitable to provide discrimination of five out of the six different pairs of the four clones, so an additional allele-specific assay was designed to distinguish between the remaining pair (clones Dd2 and D10). This new assay targeted the *msp6* gene (PF3D7_1035500), with one pair of primers specific for the Dd2 allele (5′-AAGAGCCAACATCGGAGGAATATC-3′ and 5′-CGGTTTTGAGTATTTGGTCTGGT-3′ giving an amplicon size of 116 bp) and another specific for the D10 allele (5′-ATCAATATACTGGTACATCTATATCAGGTAT-3′ and 5′-CAGAGAAGTTGTAGTACTATTACTATGAGA-3′ giving an amplicon size of 148 bp). The same thermocycling conditions, and validation of melt curves of products, were performed for each of these allele-specific qPCR assays as for the qPCR using the conserved primers described above. To validate allele-specific quantitation accuracy, mixtures of extracted parasite DNA with allelic match and mismatch were assayed in triplicate in the ratios 99:1, 90:10, 50:50, 10:90, 1:99.

*P. falciparum* clones expressing different fluorescent markers were used for microscopically counting the ratios of different parasites in mixtures, as validation tests for the accuracy of allele-specific qPCR assays for independent counting of different parasite genotypes in co-cultures. In addition to constitutively-expressed GFP fluorescent *P. falciparum* ^35^, genetically modified lines were constructed to express GFP in clone Dd2 (Dd2-GFP), and the red fluorescent protein mKate2^40^ in clone D10 (D10-mKate2). For counting of ratios of fluorescent parasites within controlled mixtures, parasite images were made on live parasites within a droplet of approximately 30% haematocrit culture under a coverslip on a glass microscope slide. These were subsequently imaged under the 40x lens of an EVOS FL Cell Imaging System, and proportions of each genotype were calculated by counting 200 parasites. For comparison of *in vitro* mixtures, ring-stage parasites were synchronised using a 5% sorbitol solution and allowed to develop into early stage trophozoite stages. Parasitaemia of the parent flask was calculated from a 1000 red blood cell count to confirm the absence of multi-nucleate schizont stages and five artificial pairwise mixed scenarios were produced consisting of 20:1, 10:1, 1:1, 1:10 and 1:20 mixtures of the two differentially fluorescent laboratory clones. Microscopic counts were performed on the two parasites expressing the different fluorophores using the alternate EVOS Texas Red and GFP Light cubes, for distinction between red and green fluorescent parasites respectively. Images of these mixtures were taken and proportions of each genotype were calculated by counting at least 200 parasites.

### Microsatellite genotyping of *ex vivo* clinical isolates

For clinical isolates, DNA underwent microsatellite genotyping at four highly polymorphic loci (TA1, Polyα, PfPK2 and TAA109), using a hemi-nested PCR protocol^41^, inner primers being labelled with particular fluorescent dyes as previously described^42^. PCR products were analysed by capillary electrophoresis using an ABI Genetic Analyzer 3730, with allele sizes discriminated using GENEMAPPER version 4.0 software, and minor alleles within an infection being scored if their peak height was at least 25% the size of the predominant allele.

## Electronic supplementary material

Supplementary Information
