# Supplementary material for: Multiplication rate variation in the human malaria parasite Plasmodium falciparum
Source: Sci Rep. 2017 Jul 25;7:6436. doi: 10.1038/s41598-017-06295-9 (PMC5527095; doi:10.1038/s41598-017-06295-9)
Supplement: Supplementary file 1 — Supplementary Information [file 41598_2017_6295_MOESM1_ESM.pdf]

Multiplication rate variation in the human malaria parasite *Plasmodium falciparum*

Supplementary Information:

Tables S1-S4

Figures S1-S3

Lee Murray [1], Lindsay B. Stewart [1], Sarah J. Tarr [1], Ambroise D. Ahouidi [2], Mahamadou Diakite [3], Alfred Amambua-Ngwa [4], David J. Conway [1]

[1] Pathogen Molecular Biology Department, London School of Hygiene and Tropical Medicine, Keppel Street, London WC1E 7HT, United Kingdom

[2] Le Dantec Hospital, Universite Cheikh Anta Diop, Dakar, Senegal

[3] Malaria Research and Training Center, University of Bamako, Mali

[4] Medical Research Council Unit, The Gambia

Table S1. Replication rates (per 48 hours) of four *P. falciparum* laboratory lines tested alone and in co-culture with heterologous lines. Parasite multiplication rate (PMR) estimates (and 95% confidence intervals) from 6-day assays as shown in Figure 2. The coefficient of determination from the data within each assay is shown (r^2).

Laboratory clones	PMR	Lower 95% CL	Upper 95 % CL	r^2
3D7 monoculture	7.13	5.67	8.96	0.97
**3D7** vs Dd2	7.11	5.44	9.29	0.96
**3D7** vs D10	7.70	6.16	9.62	0.98
**3D7** vs HB3	7.68	5.64	10.45	0.96
Dd2 Monoculture	9.13	7.02	11.87	0.98
**Dd2** vs 3D7	12.16	9.48	15.60	0.98
**Dd2** vs D10	12.39	9.98	15.37	0.99
**Dd2** vs HB3	9.92	7.46	13.18	0.97
D10 Monoculture	6.91	5.56	8.58	0.98
**D10** vs 3D7	6.76	5.31	8.61	0.97
**D10** vs Dd2	7.68	5.79	10.20	0.96
**D10** vs HB3	7.28	5.06	10.50	0.94
HB3 Monoculture	6.74	5.71	7.96	0.98
**HB3** vs 3D7	6.71	5.27	8.52	0.97
**HB3** vs Dd2	6.28	5.00	7.88	0.97
**HB3** vs D10	6.59	5.11	8.51	0.96

Table S2. Microsatellite genotypes of 11 cultured clinical isolates of *P. falciparum* successfully assayed for multiplication rates. Four loci are tabulated with PCR product allele sizes in base pairs.

Isolate ID	TA1	Poly A	PfPK2	TAA109
GUI218	166	159	172	172
GUI246	166	168, 162	166, 169	175
GUI249	163	159	166	160
MAL247	166	117	160	160
MAL250	196, 178	159	163, 156	175
SEN215	160	156	172	160
SEN220	160	153	160	160
SEN226	166	156	160	160
SEN231	166	156	169	160
SEN232	160	156	172	160
SEN251	160, 166	153, 156	169, 160	160

Table S3. Replication rates (per 48 hours) of 11 *P. falciparum* clinical isolates after a few weeks of culture *ex vivo*. Parasite multiplication rate (PMR) estimates (and 95% confidence intervals) from 4-day exponential growth assay data shown fully in Supplementary Figure S3. The coefficient of determination from the data within each assay is shown (r^2).

Isolate ID	Days *ex vivo*	PMR	Lower 95% CL	Upper 95 % CL	r^2
GUI218	32	**4.71**	3.46	6.42	0.95
GUI246	16	**5.33**	3.92	7.25	0.96
GUI249	16	**5.11**	3.39	7.71	0.93
MAL247	16	**2.51**	1.96	3.22	0.93
MAL250	16	**3.98**	3.45	4.59	0.99
SEN215	37	**5.20**	3.41	7.93	0.92
SEN220	32	**2.50**	1.83	3.40	0.88
SEN226	25	**2.46**	1.93	3.14	0.92
SEN231	18	**6.00**	4.21	8.56	0.95
SEN232	25	**3.05**	2.28	4.06	0.97
SEN251	16	**2.26**	1.85	2.76	0.93

Table S4. Replication rates (per 48 hours) of four *P. falciparum* clinical isolates that were each measured at three different time points after introduction to culture. Point estimates (and 95% confidence intervals) from 4-day growth assay data. The coefficient of determination from the data within each assay is shown (r^2).

Isolate ID	Days *ex vivo*	PMR	Lower 95% CL	Upper 95 % CL	r^2
SEN231	18	**6.00**	4.21	8.56	0.95
	67	**6.89**	5.74	8.31	0.99
	100*	**7.20**	3.80	13.65	0.95
GUI246	16	**5.33**	3.92	7.25	0.96
	45	**3.82**	3.06	4.77	0.98
	76*	**6.67**	4.26	10.44	0.97
GUI249	16	**5.11**	3.39	7.71	0.93
	45	**4.00**	3.28	4.87	0.98
	76*	**3.62**	1.67	7.86	0.84
MAL250	16	**3.98**	3.45	4.59	0.99
	45	**4.62**	3.02	7.07	0.91
	76*	**3.49**	1.95	6.25	0.90

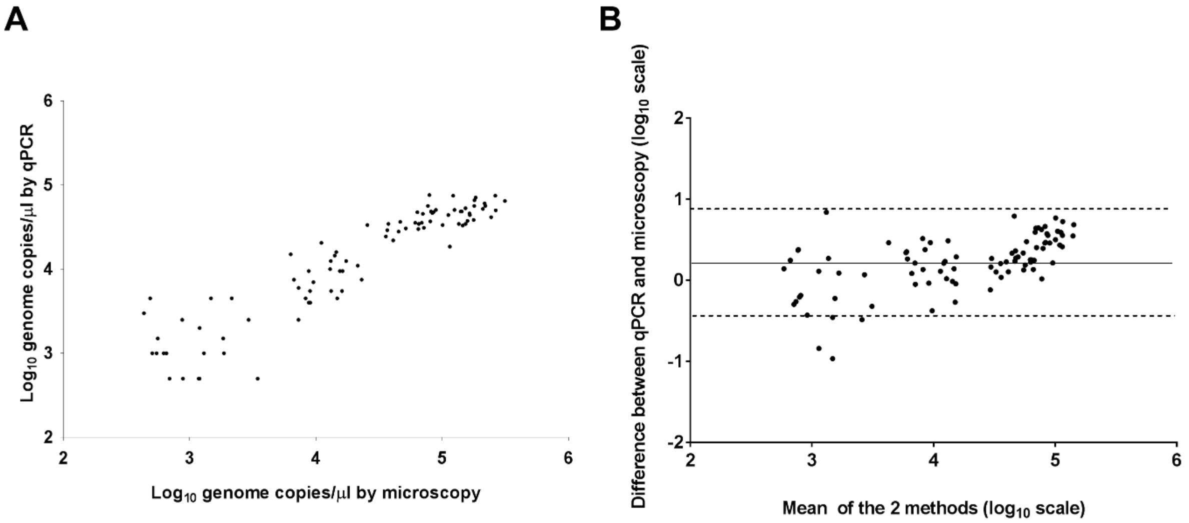

Figure S1. Comparison of parasite density measurements in culture assays measured by quantitative PCR and microscopy of Giemsa-stained thin blood films. Numbers of genome copies were estimated per microlitre of culture by qPCR as specified in the Materials and Methods. For comparison, parasitaemia was microscopically counted as proportions of infected red blood cells on a Giemsa stained smear, and genome copy estimates made by considering counted schizonts to have an average of 8 genome copies, before converting to estimated numbers of parasites per microlitre of culture at the specified haematocrit. A) Correlation plot of the two measures is highly significant from comparison of 90 measurements for four laboratory clones (Pearson's r = 0.93, p-value <0.0001****). B) Bland-Altman plot where the solid line represents the mean difference of the microscopy and the qPCR measurements and dashed lines represent 95% confidence limits of agreement between the two methods (using a mean ± 1.96 x standard deviation approach).

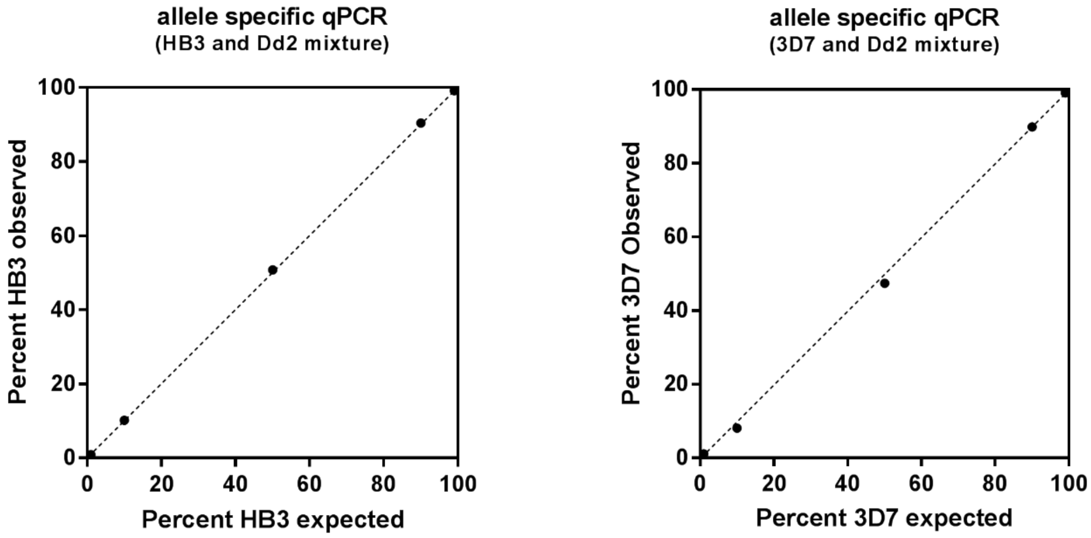

Figure S2. Allele-specificity of qPCR assays for clone-specific parasite density measurement. Each assay shown here is a comparison of observed versus expected ratios for one pair of heterologous clones. Two different assays to discriminate *msp1* alleles, used to quantify parasite DNA in heterologous mixtures shown here, have been previously described (references [21,39] in paper).

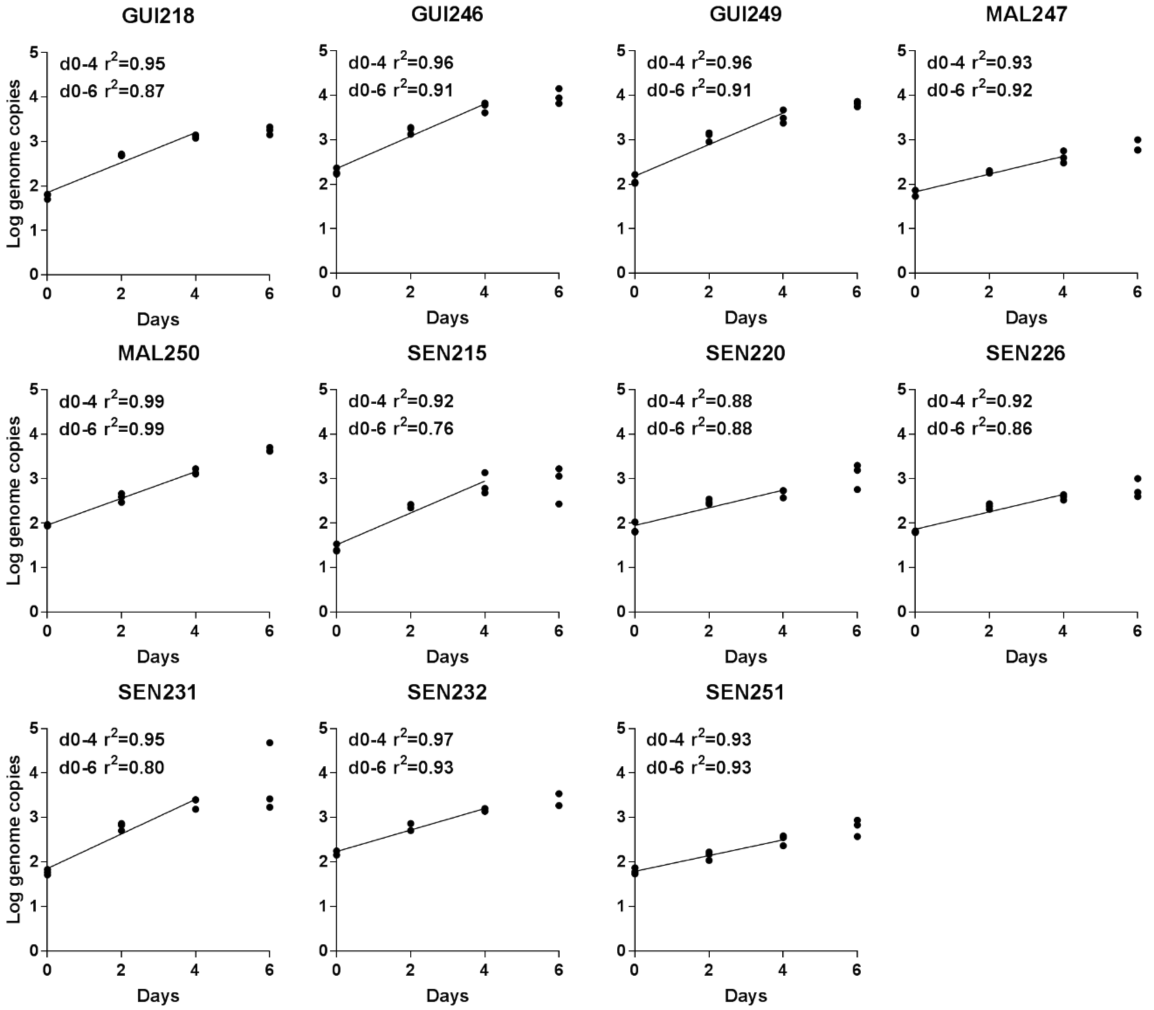

Figure S3. Multiplication rate assays of 11 clinical isolates show higher coefficients of determination for the first 4 days of growth (mean r^2 = 0.94, range from 0.88 to 0.99), than for the entire 6 days (mean r^2 = 0.89, range from 0.76 to 0.99) due to slower multiplication after 4 days for several isolates. Estimation of exponential growth was based on the day 0-4 data.
